# Functional Diversity of RNAi-Associated sRNAs in Fungi

**DOI:** 10.3390/ijms140815348

**Published:** 2013-07-24

**Authors:** Francisco E. Nicolás, Rosa M. Ruiz-Vázquez

**Affiliations:** 1 Department of Genetics and Microbiology, Faculty of Biology, University of Murcia, Murcia 30100, Spain; E-Mail: rmruiz@um.es; 2 Regional Campus of International Excellence “Campus Mare Nostrum”, Murcia 30100, Spain

**Keywords:** fungi, sRNA, siRNA, masiRNAs, esRNA, ex-siRNA, milRNA, qiRNA

## Abstract

Yeast and filamentous fungi have been essential model systems for unveiling the secrets of RNA interference (RNAi). Research on these organisms has contributed to identifying general mechanisms and conserved eukaryotic RNAi machinery that can be found from fungi to mammals. The development of deep sequencing technologies has brought on the last wave of studies on RNAi in fungi, which has been focused on the identification of new types of functional small RNAs (sRNAs). These studies have discovered an unexpected diversity of sRNA, biogenesis pathways and new functions that are the focus of this review.

## 1. Introduction

RNAi is a negative regulatory mechanism that represses the expression of target RNAs. It was firstly described as a mechanism triggered by double-stranded RNA (dsRNA) in the worm *Caenorhabditis elegans* [1]. However, this phenomenon was previously discovered in plants and fungi, in which it was named with the terms co-suppression and quelling, respectively [2,3]. Both in plants and fungi, it was observed after transforming a wild type strain with exogenous gene sequences that are required for the biosynthesis of different pigments. The result of these transformations was the lack of expression of both the transgene and the endogenous homolog sequence, which produced an albino phenotype instead of the expected overproduction of pigments [3]. In the case of fungi, these experiments were carried out in *Neurospora crassa*, a robust study model with many available genetic tools that allowed the cloning of several genes involved in RNAi and helped to unveil the main core of the RNAi machinery. Using a transformed strain that showed a stable albino phenotype for mutagenesis, several quelling deficient mutants (*qde*) were isolated [4]. Complementation analyses of these mutants concluded in the identification of three different genes: *qde-1*, *qde-2* and *qde-3*. An RNA dependent RNA polymerase (RDRP), encoded by *qde-1*, was the first component of the RNAi machinery that was cloned in these screenings [4]. The role of this enzyme is to generate dsRNA from the aberrant RNAs (aRNAs) that are hypothetically produced from the transgene. The essential role of dsRNA as the main trigger of RNAi was demonstrated soon after in *C. elegans* in a wonderful work that was awarded the Nobel prize in 2006 [1]. The next enzyme required in the RNAi pathway is a ribonuclease type III that processes the dsRNA into the small interfering RNAs (siRNAs), a special type of sRNAs that exhibits a fixed size between 19 and 25 nt and has 5′ phosphates and two nucleotide overhangs on the 3′ ends [5]. This ribonuclease is known as the Dicer enzyme and despite its essential role in RNAi, it was not one of the three *qde* genes that were initially identified in this fungus [6]. This was due to the partially redundant activity of the two Dicer-like proteins that were found later in *N. crassa*, when the genome sequence was available [6]. The third essential enzyme of the RNAi core machinery is the Argonaute protein (Ago) which is encoded by *qde-2* gene [4]. The Ago protein acts downstream of Dicer, incorporating the siRNAs into the so-called RNA-induced silencing complex (RISC), a protein complex containing Ago, the siRNA, and other accessory proteins. The slicing activity of Ago produces a nicked siRNA duplex, which helps the exonuclease QIP to remove the passenger strand of the siRNA duplex, provoking the activation of the RISC complex [7,8]. Once the RISC complex has been activated, it uses the guide strand of the siRNA to identify complementary mRNA molecules, which are degraded by endonucleolytic and exonucleolytic cleavages. The last quelling deficient mutant that was cloned in *N. crassa*, *qde-3*, encodes a RecQ DNA helicase which could be involved in the generation of aRNAs/dsRNA [4]. Mutants in the *qde-3* gene present defective mechanisms of both RNAi and DNA repair in *N. crassa* [9,10]. It has been suggested that QDE-3 could be involved in the recognition of exogenous DNA and the generation of aRNA by recruiting QDE-1 to the ssDNA resulting from aberrant DNA structures, which could be produced during replication or recombination of repetitive sequences [11]. The synthesis of the aRNA has been traditionally assigned to RNA polymerase II, which would transcribe the ssDNA template to produce the aRNA used by QDE-1 to generate dsRNA. However, the crystal structure of QDE-1 has revealed that its catalytic core is structurally similar to eukaryotic DNA-dependent RNA polymerases (DdRPs) rather than viral RdRPs [11,12]. These finding suggested that QDE-1 could be both an RdRP and a DdRP, being required both for the synthesis of the aRNA and the subsequent dsRNA production; this dual activity of QDE-1 has been experimentally demonstrated [11].

The RNAi mechanism and its canonical pathway were initially described as a host defense mechanism that protects the genome from invasive nucleic acids, such as viruses and transposons. This defensive role supports that RNAi is an essential mechanism that has been evolutionary conserved through the entire eukaryotic domain. However, the existence of several eukaryotic microbes that lack an active RNAi pathway raises the question of how they can survive without the protective role of RNAi. It has been proposed that the RNAi mechanism may represent a liability rather than an advantage in some special evolutionary scenarios, forcing the selection of RNAi-deficient species [13,14]. This is the case of several yeast species, including *Saccharomyces cerevisiae*, in which the absence of functional RNAi has been correlated with the presence of endemic dsRNA viruses that confer selective advantages to the host cells [13]. Besides the defensive role of RNAi, the discovery in recent years of several new pathways that are endogenously triggered has unveiled RNAi as a new regulatory mechanism that controls different cell functions. The different fungal sRNAs related to both aspects of RNAi, host defense and regulation of gene expression, are the focus of this review (Table 1).

## 2. Host Defense sRNAs in Fungi

The RNAi mechanism can be triggered by a wide variety of exogenous nucleic acids that represent a threat for genome integrity. Thus, different exogenous nucleic acids such as integrative transgenes, plasmids, viruses and transposons have been found to trigger the RNAi mechanism against whatever is transcribed from them. All the pathways and different types of sRNAs that are produced during this response are reviewed in this section.

### 2.1. siRNAs Triggered by Randomly Integrated Exogenous Sequences

The first type of sRNAs found in fungi were siRNAs produced to silence exogenous sequences that are homologous to an endogenous gene [7]. In particular, these siRNAs were found in *N. crassa* after the introduction of exogenous sequences, which were randomly integrated at ectopic locations of the genome of this fungus [7]. Wild type mycelium of *N. crassa* shows a bright orange phenotype due to the synthesis of carotenoids. Mutations in any of the three structural genes involved in the carotenoid biosynthesis, *albino-1 (al-1)*, *albino-2 (al-2)* and *albino-3 (al-3)*, result in an easily detectable albino phenotype. Transformation of the wild type strain of *N. crassa* with different constructs of the *al-1* gene, encoding the phytoene dehydrogenase gene, produced 36% albino transformants [3]. The mechanism that initiates the production of these siRNAs is still unclear. They are processed from dsRNA, as it is a common step for all the sRNAs associated to RNAi. The production of this dsRNA from the transgene and how the cell distinguishes between the endogenous gene and the exogenous transgene to initiate the RNAi mechanism are not certainly known. The analysis of these albino transformants has revealed that the copy number of the integrated exogenous sequences ranged from 2 to 20, with no gene rearrangements in any of the analyzed transformants that could explain the production of dsRNA. The analysis of the revertants, which are albino transformants that turn to wild type in the following generation, showed a reduced copy number of exogenous sequences, although this cannot be the direct reason of the albino phenotype, as indicated by the existence of albino transformants containing only two copies of the transgene. The current most accepted hypothesis to explain the early steps in the biogenesis of transgene-induced siRNAs is the production of aRNA from the repetitive transgene sequences integrated into the genome (see Introduction). Besides *N. crassa*, RNAi triggered by the integration of exogenous sequences has been described in others fungi such as *Cladosporium fulvum* and *Schizophyllum commune* [24,25].

### 2.2. Two Classes of siRNAs Triggered by Non-Integrative Transgenes in *Mucor circinelloides*

*M. circinelloides* is an outstanding study model among zygomycetes due to the existence of an efficient transformation protocol that allows genetics analysis in this fungus [26]. An added value of genetic transformation of *M. circinelloides* is that a non-integrative transgene can be delivered in self replicative plasmids. These plasmids behave as extrachromosomal DNA molecules inside the nucleus of this fungus. They exhibit all the features of self-replicative molecules, such as a high frequency of transformation, mitotic instability, easy re-isolation in a non-modified state from undigested transformant DNA and detection as discrete DNA molecules in Southern hybridization experiments [27]. An advantage of triggering RNAi using this kind of plasmid is that the expression of the non-integrative transgene is not affected by position effects or host regulatory sequences at insertion sites, such as inverted promoters or repeated transgene integrations in different orientations. This clean system to trigger RNAi enables a correlation between the transgene copy number and the strength of the silenced phenotype in *M. circinelloides,* and added new evidence supporting the hypothesis of aRNA as the link molecule between the invasive DNA and the necessary dsRNA [15]. However, the most interesting feature of RNA silencing in *M. circinelloides* is the existence of two different classes of antisense siRNAs that are differentially accumulated during vegetative growth. A long siRNA of 25 nt is more abundant at the beginning of the growth cycle, whereas a shorter 21 nt siRNA is accumulated at the end of the vegetative cycle and transmitted to the next generation through the spore [15]. The biological function of these two different classes of siRNAs and the differential role that they could play during growth and development is still unknown.

### 2.3. Transposon Control siRNAs

The mechanism of RNAi was initially observed after the integration of repetitive transgenes in the genome of plant and fungal cells [2,3]. It is obvious that this complex mechanism has not evolved to protect the genome against transgenes that are artificially delivered into the cell by a laboratory protocol. However, the repetitive nature of transgene integration in silenced strains immediately suggested that RNAi could be a defense mechanism against invasive repetitive sequences like transposons. There are numerous examples through the eukaryotic kingdom showing the important role of RNAi in the maintenance of genome integrity by silencing these transposable elements [28]. The suggested mechanism proposes fortuitous transcription of the terminal inverted repeats of the transposon, which leads to the formation of dsRNA and the consequent activation of RNAi [29]. In fungi, genetic analysis with an African strain of *N. crassa* that harbors a LINE-like transposon showed that the RNAi mechanism is required to suppress transposon replication [16]. This analysis revealed that QDE-2 and Dicer, but not QDE-1 and QDE-3, are essential for silencing the transposable elements and suggested that transposons trigger the RNAi mechanism through the direct synthesis of dsRNA from inverted repeats generated by transposition. Later, the role of siRNAs in transposition control has been demonstrated in other fungi. The introduction of the LTR-retrotransposon MAGGY into *Magnaporthe oryzae* strains harboring mutations in the *dicer* genes resulted in a deficient siRNA accumulation, higher MAGGY mRNA production and faster MAGGY copy number increase than did the wild-type. These results indicated that RNA silencing functioned as an effective defense mechanism against transposable elements [30]. Moreover, deep sequencing of small RNA molecules has identified siRNAs derived from transposable sequences in *M. circinelloides* [20] and *M. oryzae* [31].

### 2.4. Antiviral siRNAs

Defense against invasive viruses along with the control of transposable elements were the first functions associated to RNAi. This defensive role has been identified in a diverse range of distant eukaryotes, including plants [32], worms [33], flies [34] and mammals [35]. All these organisms produce specific siRNAs that target and destroy the invasive viral genome. In fungi, the defensive action of siRNAs against mycoviruses was experimentally demonstrated in the chestnut blight fungus *Cryphonectria parasitica*, in which one of the two dicer-like genes, *dcl2*, and only one of the four argonaute-like genes, *agl2*, are required for antiviral defense response [17,36]. Mutants in one of these two genes lack the ability to avoid viral infections, becoming debilitated strains that are highly susceptible to mycovirus infections and present a hypovirulent phenotype when they infect their host [37]. The role of these two genes in the viral defense mechanism is similar to other RNAi pathways; the Dicer protein produces virus-specific siRNAs that are transferred to Argonaute containing complexes that target and destroy viral sequences. In response to this RNAi-based viral immunity system, viruses have developed RNAi suppression mechanisms. This is the case for the hypovirus CHV1-EP713, a mycovirus that infects *C. parasitica,* and that has developed an RNAi suppression mechanism based on the protein p29. This protein is a RNAi suppressor that acts in a promoter-dependent manner, mediating the repression of an *argonaute*-like gene (*agl2*) and therefore blocking the activation of the RNAi based antiviral response [38]. Similarly, *Aspergillus nidulans* exhibits a fully functional RNAi pathway whose efficiency is suppressed after mycoviruse infections, which confirms the existence of an RNAi suppressor encoded by the virus and the antiviral purpose of the RNAi machinery [39].

### 2.5. siRNAs Associated with Meiotic Silencing by Unpaired DNA

Meiotic silencing by unpaired DNA (MSUD) is a genome surveillance system identified in *N. crassa* and *Gibberella zeae* that transiently silences genes unpaired during the pairing stage of the meiotic prophase I, along with any other DNA homologous to the unpaired sequences [40,41]. This system protects the genome from any trouble that could represent unpaired DNA segments, such as viruses and transposons on the move. The mechanism of action is similar to other RNAi pathways, thus, an aRNA is transcribed from unpaired DNA regions and this aRNA is used as a template to synthesize dsRNA, which is processed into MSUD-associated small interfering RNAs (masiRNAs) [18]. However, the machinery involved in this process presents some differences when compared with the canonical RNAi pathway. For instance, two new components, *sad-1* (*suppressor of ascus dominance 1*) and *sad-2*, have been identified in MSUD [40–42]. The first gene, *sad-1*, encodes an RdRP that is a paralog of *qde-1* [40,43]. SAD-2 is a new element in this pathway that does not contain any conserved domains and that is required for the proper localization of SAD-1 [42]. DCL-1 and QIP are common elements shared between MSUD and canonical RNAi pathways, but the Argonaute protein is encoded by *sms-2* (*suppressor of meiotic silencing 2*) in MSUD. Another element required for MSUD is SAD-3, a putative RNA/DNA helicase that is homologous to *S. pombe* Hrr1. The protein Hrr-1 is required for RNAi-mediated heterochromatin formation in fission yeast, which suggests that heterochromatinization and MSUD could be two processes that are mechanistically related [44,45].

### 2.6. Sex Induced siRNAs in *Cryptococcus neoformans*

The human fungal pathogen *C. neoformans* has the usual RNAi machinery with Argonaute, Dicer and RdRP as the central components, similarly to other fungi in which RNAi has been described. However, in this fungus the induction of RNAi by tandem integration of transgenes results in a special sex-induced silencing (SIS), as the repetitive transgene is silenced at an ~250-fold lower frequency during vegetative mitotic growth compared with sexual reproduction [19]. Regular siRNAs have been found by deep sequencing during SIS, but the most interesting result is that along with siRNAs from the transgene, many other siRNAs from transposons were identified. The production of these siRNAs was impaired in *rdrp* mutant strains in which the expression of a group of retrotransposons was notably increased during mating. The consequence of this uncontrolled transposon activity was a higher transposition/mutation rate. The interpretation of these results suggests that the function of siRNAs during SIS is to protect the genome of the progeny by reducing transposon activity during the sexual cycle [19].

## 3. Regulatory Endogenous sRNAs in Fungi

Endogenous short RNAs (esRNAs) are similar to siRNAs in their biogenesis, with the main difference based in the fact that they are directly produced from an endogenous precursor rather than an exogenous trigger molecule. Most classes of esRNAs are produced from precursor double-stranded RNAs, which are directly transcribed from the genome or generated after the action of RdRP enzymes. As it happens with exogenously triggered siRNAs, esRNAs are usually processed by a member of the Dicer family and are also incorporated into an effector complex containing a member of the Argonaute family, similar to the RISC complex. The most relevant example of esRNAs were found in animals and plants, the so called microRNAs (miRNAs), a class of esRNAs that are produced from hairpin structured RNAs and target mRNAs for their repression [46]. miRNAs play a role in diverse processes such as development, cell differentiation, adaptation to environmental changes and disease [46]. For a long time, miRNAs have been widely considered to be absent in fungi, although *dicer* mutants of several fungi have been reported to be affected in vegetative and developmental processes [47], suggesting the existence of esRNAs with regulatory functions in this kingdom. The application in fungi of the new deep sequencing technologies has revealed the existence of several new regulatory esRNAs that are described in this section.

### 3.1. Exonic-siRNAs in *M. circinelloides*

The exonic-siRNAs (ex-siRNAs) were the first esRNAs found in fungi that regulate the expression of endogenous target genes through the repression of the corresponding mRNA [20]. These ex-siRNAs were discovered in the basal fungus *M. circinelloides* in which previous results had suggested a role of the RNAi machinery in the regulation of several processes such as asexual sporulation, vegetative development and hyphal morphology [47,48]. Deep sequencing of the short RNA content in the wild type and several RNAi mutants of this fungus showed the existence of ex-siRNAs as a new type of esRNAs. They are produced from exons of the same genes that are later regulated through the repression of the corresponding mRNA [20]. Hundreds of ex-siRNAs-producing exons have been identified, which correspond to a total of 276 genes, since some genes contain more than one exon producing these ex-siRNAs. There are four different classes of ex-siRNAs (classes 1–4) that have been classified based on the differential RNAi machinery involved in their biogenesis. The first two classes (classes 1 and 2) include all ex-siRNAs that are DCL2-dependent and present a strong preference for uracil (92%) in the first position of the molecule, a preference that is shared among Argonaute-bound guide RNAs of animal, plants and other fungi. The ex-siRNAs belonging to class 2 showed reduced levels in the *rdrp1^−^* mutant but not in the *rdrp2^−^* strain, which specifically defines this class. Class 1 contains a few ex-siRNAs that do not require RdRP1 but most of them depend on RdRP2. Class 3 covers a significant group of ex-siRNAs that are processed either by DCL1 or DCL2, since they are down-regulated only in the double *dicer* mutant but not in *dcl1^−^* or *dcl2^−^* single mutants, indicating a redundant function of the two *dicer* genes in the production of this class of ex-siRNAs. Oppositely, both RdRP enzymes are required for the biogenesis of class 3, as these ex-siRNAs are down-regulated in mutants of either *rdrp1* or *rdrp2*. The class 4 contains ex-siRNAs produced from only five exons and they are down-regulated in *dcl1^−^* but not in *dcl2^−^*. One of the exons included in this class encodes a conserved protein that is involved in polarized growth (at the tip of the hypha), along with other proteins involved in the mitochondria metabolism and ribosome function, which could explain the abnormal hyphal morphology and lower growth rate described in *dcl1^−^* mutants [47]. All ex-siRNAs are down regulated in the *ago-1^−^* mutant, although only those of classes 1 and 2 are specifically bound to the Ago-1 protein, revealing the complexity of the esRNA biogenesis pathways in fungi [49].

### 3.2. MicroRNA-like RNAs in *N. crassa*

Analysis of esRNAs associated with the *N. crassa* QDE-2 protein identified several types of esRNAs, including those that share some similarities with conventional miRNAs from animals and plants [21]. These miRNA-like RNAs (milRNAs) are produced from stem-loop RNA precursors and most of them require Dicer in their biogenesis. This analysis also suggested that milRNAs could target endogenous RNA transcripts with imperfect complementarity, like regular miRNAs do. The biogenesis of these milRNAs, as with ex-siRNAs in *M. circinelloides*, shows a diverse use of the components of the RNAi machinery in order to produce four different classes of milRNAs (milR-1, milR-2, milR-3 and milR-4). The production of the first type of milRNA, milR-1, is completely dependent on Dicers, QDE-2 (but not its catalytic activity) and the exonuclease QIP. milR-2 milRNAs do not depend on Dicers but require QDE-2 and its catalytic activity. The production of milR-3 miRNAs only depends on Dicers activity, being the most similar pathway to plants miRNA synthesis. The biogenesis of the last milRNA class, milRNA-4, suggests the involvement of an unknown nuclease, as it is only partially dependent on Dicers. Reconstitution of the QDE-2-dependent milR-1 biogenesis *in vitro* has revealed the role of the RNA exosome, a 3′ to 5′ exonuclease complex, in determining the size of milR-1, demonstrating the importance of the exosome in esRNA processing [50]. Unlike in plants and animals, in which miRNAs are produced by RNA Pol II, the four major types of milRNAs identified in *N. crassa* are transcribed by the RNA Pol III, although Pol II was found to be associated with some milR loci, suggesting collaboration between the two polymerases in milRNAs production [51].

### 3.3. Regulatory esRNAs in *Magnaporthe oryzae*

The third study that analyzed the esRNAs content in a fungus by deep sequencing was carried out in *M. oryzae*, a model organism for the study of pathogen-host interactions in plants [31]. In this study, conversely to *N. crassa*, no putative miRNAs were found, and similarly to *M. circinelloides*, a profile composed of protein coding genes, intergenic regions and repetitive elements derived esRNAs was described. Interestingly, there were differences between the spectrum of esRNAs accumulated in vegetative and specialized-infection tissues. Whereas the esRNAs accumulated in vegetative mycelia were enriched for sequences that mapped to transposable elements, tRNA-derived fragments (tRFs) were the most abundant sRNA species identified in the appressorium, a specialized hypha that is involved in the invasion of the host plant cell. The specific presence of tRFs in the appresoria has been proposed to be part of a mechanism that restricts protein biosynthesis in order to direct cellular metabolism towards infection [31]. However, the biogenesis of tRFs is still unknown and there is no experimental evidence on the involvement of the RNAi machinery in their production, suggesting that they cannot be considered as *bona fide* esRNAs.

### 3.4. siRNA-Mediated Regulation of Heterochromatin in *Schizosaccharomyces pombe*

Regulation of heterochromatin formation depends on the components of the RNAi pathway in *S. pombe* [22]. Heterochromatin is transcriptionally inactive DNA with a highly condensed structure that can be found at three different regions of the *S. pombe* genome: centromeres, telomeres and mating-type loci. Some of these regions can lose their condensed structure and become transcriptionally active. Later, the re-heterochromatinization of these regions occurs in an RNAi-dependent manner and is mediated by the production of specific siRNAs. The mechanism of RNAi in transcriptional silencing uses a similar machinery core to the post-transcriptional gene silencing mechanism. Thus, an RdRP is required to produce dsRNA from the nascent transcripts that are synthesized by RNA Pol II during the S phase of the cell cycle. The dsRNA is processed by Dicer into siRNAs that are then loaded onto *Ago1* in the so called RNA-induced transcriptional silencing (RITS) complex [52]. One difference between RITS and RISC is the presence in RITS of Chp1, a chromodomain-containing protein that binds the histone H3 [53]. Besides the binding properties of Chp1, RITS uses the guide strand to target the nascent transcript and reinforce the interaction with the region that is initiating heterochromatinization. Recruitment of RITS to these regions allows it to interact with other chromatin-modifying components, forming a major protein complex that spreads heterochromatinization through the region. In this interaction, RITS associates with the RNA-directed RNA polymerase complex (RDRC), a protein complex which contains an RNA polymerase, Rdrp1, a putative helicase termed Hrr1, and Cid12, a member of the Trf4 and Trf5 family [45]. The association between RITS and RDRC is siRNA and Clr4 dependent, which suggests that this association requires histone H3K9 methylation and occurs by a mechanism that involves tethering the nascent transcript to the chromatin. Several lines of evidence suggest that the siRNA guides RITS to target the nascent RNA transcript rather than the DNA complement strand [54]. This initiates the synthesis of dsRNA from the nascent transcript by the Rdrp1 present in RDRC, which leads to the processing of the resultant dsRNA by Dicer at the same location, as the Dicer enzyme is also required for the association between RITS and RDRC [45,55]. The activity of Dicer produces new siRNAs that amplifies the mechanism and helps to maintain and spread the heterochromatinization.

### 3.5. QDE-2-Interacting sRNAs Induced by DNA Damage

QDE-2-interacting sRNAs (qiRNAs) are a special type of esRNAs identified in *N. crassa* that are produced after treating this fungus with DNA-damaging agents [23]. The basic structure and biogenesis of qiRNAs are quite similar to regular siRNAs that are found interacting with Argonaute proteins. Thus, qiRNAs are 21–23 nt long, they require RdRP, Dicer and Argonaute proteins for their biogenesis, as well as the QDE-3 helicase, and they usually exhibit a 5′ uridine, like regular siRNAs. The special feature of qiRNAs is their production from repetitive sequences of rDNA as a response to DNA damage. It was suggested that the triggering signal that activates the production of qiRNA is the synthesis of aRNA from damaged DNA sequences, in which double-stranded breaks and replication stress induce the formation of aberrant DNA structures. However, genetic screens designed to identify genes required for qiRNA biogenesis have revealed that homologous recombination is the only process required for qiRNA production, suggesting that *N. crassa* utilizes homologous recombination triggered by DNA damage to identify repetitive DNA loci [56]. The proposed model suggests that DNA damage promotes the formation of aberrant forms of recombination intermediates of repetitive DNA, which are recognized by QDE-3 and QDE-1 to produce aRNA and dsRNA [56]. The same mechanism has been suggested to operate in the initiation of silencing by repetitive transgenes, since homologous recombination is also required for quelling in *N. crassa*, indicating that quelling and qiRNA production share a common mechanism [56].

## 4. Conclusions

The kingdom Fungi has deeply contributed to our understanding of RNAi and its functions. Initially discovered as a defense mechanism against transposon and viral invasion, the RNAi mechanism has now emerged as a complex mechanism of gene regulation. Recent discoveries show several new sRNAs and different biogenesis pathways involved in a high diversity of new functions associated to the RNAi mechanism. Future studies on RNAi in fungi will reveal the whole picture of the role of these new regulatory sRNAs, enlightening the evolutionary origin of RNAi in eukaryotes.

## Figures and Tables

**Table 1:** Classes of RNAi-dependent sRNAs in fungi.

Function	Name	Acronym	Inducer	Firstly described	Reference
Host Defense	Small Interfering RNAs	siRNAs	Integrative transgenes	*Neurospora crassa*	[7]
			Non integrative transgenes	*Mucor circinelloides*	[15]
			Transposons	*Neurospora crassa*	[16]
			Viruses	*Cryphonectria parasitica*	[17]

	MSUD-associated small interfering RNAs	masiRNAs	Unpaired DNA	*Neurospora crassa*	[18]

	Sex Induced Silencing siRNAs	SIS siRNAs	Repetitive transgenes	*Cryptococcus neoformans*	[19]

Endogenous Gene Regulation	Exonic-siRNAs	ex-siRNA	Regular transcription	*Mucor circinelloides*	[20]

	MiRNA-like RNAs	milRNAs	Regular transcription	*Neurospora crassa*	[21]

	Heterochromatin derived siRNAs	siRNAs	Heterochromatin transcription	*Schizosaccharomyces pombe*	[22]

	QDE-2-interacting sRNAs	qiRNAs	DNA damage	*Neurospora crassa*	[23]

## References

[1] Fire A., Xu S., Montgomery M.K., Kostas S.A., Driver S.E., Mello C.C. (1998). Potent and specific genetic interference by double-stranded RNA in *Caenorhabditis elegans*. Nature.

[2] Napoli C., Lemieux C., Jorgensen R. (1990). Introduction of a chimeric chalcone synthase gene into petunia results in reversible co-suppression of homologous genes in trans. Plant Cell.

[3] Romano N., Macino G. (1992). Quelling, transient inactivation of gene expression in *Neurospora crassa* by transformation with homologous sequences. Mol. Microbiol.

[4] Cogoni C., Macino G. (1997). Isolation of quelling-defective (qde) mutants impaired in posttranscriptional transgene-induced gene silencing in *Neurospora crassa*. Proc. Natl. Acad. Sci. USA.

[5] Bernstein E., Caudy A.A., Hammond S.M., Hannon G.J. (2001). Role for a bidentate ribonuclease in the initiation step of RNA interference. Nature.

[6] Catalanotto C., Pallotta M., ReFalo P., Sachs M.S., Vayssie L., Macino G., Cogoni C. (2004). Redundancy of the two dicer genes in transgene-induced posttranscriptional gene silencing in *Neurospora crassa*. Mol. Cell. Biol.

[7] Catalanotto C., Azzalin G., Macino G., Cogoni C. (2002). Involvement of small RNAs and role of the *qde* genes in the gene silencing pathway in *Neurospora*. Genes Dev.

[8] Maiti M., Lee H.C., Liu Y. (2007). QIP; a putative exonuclease; interacts with the *Neurospora* Argonaute protein and facilitates conversion of duplex siRNA into single strands. Genes Dev.

[9] Cogoni C., Macino G. (1999). Posttranscriptional gene silencing in *Neurospora* by a RecQ DNA helicase. Science.

[10] Pickford A., Braccini L., Macino G., Cogoni C. (2003). The QDE-3 homologue RecQ-2 co-operates with QDE-3 in DNA repair in *Neurospora crassa*. Curr. Genet.

[11] Lee H.C., Aalto A.P., Yang Q., Chang S.S., Huang G., Fisher D., Cha J., Poranen M.M., Bamford D.H., Liu Y. (2010). The DNA/RNA-dependent RNA polymerase QDE-1 generates aberrant RNA and dsRNA for RNAi in a process requiring replication protein A and a DNA helicase. PLoS Biol.

[12] Laurila M.R., Salgado P.S., Makeyev E.V., Nettelship J., Stuart D.I., Grimes J.M., Bamford D.H. (2005). Gene silencing pathway RNA-dependent RNA polymerase of *Neurospora crassa*, yeast expression and crystallization of selenomethionated QDE-1 protein. J. Struct. Biol.

[13] Drinnenberg I.A., Fink G.R., Bartel D.P. (2011). Compatibility with killer explains the rise of RNAi-deficient fungi. Science.

[14] Nicolas F.E., Torres-Martínez S., Ruiz-Vázquez R.M. (2013). Loss and retention of RNA interference in fungi and parasites. PLoS Pathog.

[15] Nicolas F.E., Torres-Martinez S., Ruiz-Vazquez R.M. (2003). Two classes of small antisense RNAs in fungal RNA silencing triggered by non-integrative transgenes. EMBO J.

[16] Nolan T., Braccini L., Azzalin G., de Toni A., Macino G., Cogoni C. (2005). The post-transcriptional gene silencing machinery functions independently of DNA methylation to repress a LINE1-like retrotransposon in *Neurospora crassa*. Nucleic Acids Res.

[17] Zhang X., Segers G.C., Sun Q., Deng F., Nuss D.L. (2008). Characterization of hypovirus-derived small RNAs generated in the chestnut blight fungus by an inducible DCL-2-dependent pathway. J. Virol.

[18] Hammond T.M., Xiao H., Boone E.C., Decker L.M., Lee S.A., Perdue T.D., Pukkila P.J., Shiu P.K.T. (2013). Novel proteins required for meiotic silencing by unpaired DNA and siRNA generation in *Neurospora crassa*. Genetics.

[19] Wang X., Hsueh Y.P., Li W., Floyd A., Skalsky R., Heitman J. (2010). Sex-induced silencing defends the genome of *Cryptococcus neoformans* via RNAi. Genes Dev.

[20] Nicolas F.E., Moxon S., de Haro J.P., Calo S., Grigoriev I.V., Torres-Martinez S., Moulton V., Ruiz-Vázquez R.M., Dalmay T. (2010). Endogenous short RNAs generated by Dicer 2 and RNA-dependent RNA polymerase 1 regulate mRNAs in the basal fungus *Mucor circinelloides*. Nucleic Acids Res.

[21] Lee H.C., Li L., Gu W., Xue Z., Crosthwaite S.K., Pertsemlidis A., Lewis Z.A., Freitag M., Selker E.U., Mello C.C. (2010). Diverse pathways generate microRNA-like RNAs and Dicer-independent small interfering RNAs in fungi. Mol. Cell.

[22] Volpe T.A., Kidner C., Hall I.M., Teng G., Grewal S.I., Martienssen R.A. (2002). Regulation of heterochromatic silencing and histone H3 lysine-9 methylation by RNAi. Science.

[23] Lee H.C., Chang S.S., Choudhary S., Aalto A.P., Maiti M., Bamford D.H., Liu Y. (2009). qiRNA is a new type of small interfering RNA induced by DNA damage. Nature.

[24] Hamada W., Spanu P.D. (1998). Co-suppression of the hydrophobin gene HCf-1 is correlated with antisense RNA biosynthesis in *Cladosporium fulvum*. Mol. Gen. Genet.

[25] Schuurs T.A., Schaeffer E.A., Wessels J.G. (1997). Homology-dependent silencing of the SC3 gene in *Schizophyllum commune*. Genetics.

[26] Gutierrez A., Lopez-Garcia S., Garre V. (2011). High reliability transformation of the basal fungus *Mucor circinelloides* by electroporation. J. Microbiol. Methods.

[27] Roncero M.I., Jepsen L.P., Stroman P., van Heeswijck R. (1989). Characterization of a leuA gene and an ARS element from *Mucor circinelloides*. Gene.

[28] Buchon N., Vaury C. (2006). RNAi, a defensive RNA-silencing against viruses and transposable elements. Heredity.

[29] Sijen T., Plasterk R.H. (2003). Transposon silencing in the *Caenorhabditis elegans* germ line by natural RNAi. Nature.

[30] Murata T., Kadotani N., Yamaguchi M., Tosa Y., Mayama S., Nakayashiki H. (2007). siRNA-dependent and -independent post-transcriptional cosuppression of the LTR-retrotransposon MAGGY in the phytopathogenic fungus *Magnaporthe oryzae*. Nucleic Acids Res.

[31] Nunes C.C., Gowda M., Sailsbery J., Xue M., Chen F., Brown D.E., Oh Y.Y., Mitchell T.K., Dean R.A. (2011). Diverse and tissue-enriched small RNAs in the plant pathogenic fungus; *Magnaporthe oryzae*. BMC Genomics.

[32] Harvey J.J., Lewsey M.G., Patel K., Westwood J., Heimstadt S., Carr J.P., Baulcombe D.C. (2011). An antiviral defense role of AGO2 in plants. PLoS One.

[33] Wilkins C., Dishongh R., Moore S.C., Whitt M.A., Chow M., Machaca K. (2005). RNA interference is an antiviral defence mechanism in *Caenorhabditis elegans*. Nature.

[34] Zambon R.A., Vakharia V.N., Wu L.P. (2006). RNAi is an antiviral immune response against a dsRNA virus in *Drosophila melanogaster*. Cell. Microbiol.

[35] Jeang K.T. (2012). RNAi in the regulation of mammalian viral infections. BMC Biol.

[36] Sun Q., Choi G.H., Nuss D.L. (2009). A single Argonaute gene is required for induction of RNA silencing antiviral defense and promotes viral RNA recombination. Proc. Natl. Acad. Sci. USA.

[37] Zhang X., Nuss D.L. (2008). A host dicer is required for defective viral RNA production and recombinant virus vector RNA instability for a positive sense RNA virus. Proc. Natl. Acad. Sci. USA.

[38] Segers G.C., van Wezel R., Zhang X., Hong Y., Nuss D.L. (2006). Hypovirus papain-like protease p29 suppresses RNA silencing in the natural fungal host and in a heterologous plant system. Eukaryot. Cell.

[39] Hammond T.M., Andrewski M.D., Roossinck M.J., Keller N.P. (2008). *Aspergillus* mycoviruses are targets and suppressors of RNA silencing. Eukaryot. Cell.

[40] Shiu P.K., Raju N.B., Zickler D., Metzenberg R.L. (2001). Meiotic silencing by unpaired DNA. Cell.

[41] Son H., Min K., Lee J., Raju N.B., Lee Y.W. (2011). Meiotic silencing in the homothallic fungus *Gibberella zeae*. Fungal Biol.

[42] Shiu P.K., Zickler D., Raju N.B., Ruprich-Robert G., Metzenberg R.L. (2006). SAD-2 is required for meiotic silencing by unpaired DNA and perinuclear localization of SAD-1 RNA-directed RNA polymerase. Proc. Natl. Acad. Sci. USA.

[43] Shiu P.K., Metzenberg R.L. (2002). Meiotic silencing by unpaired DNA, properties; regulation and suppression. Genetics.

[44] Hammond T.M., Xiao H., Boone E.C., Perdue T.D., Pukkila P.J., Shiu P.K. (2011). SAD-3, a putative helicase required for meiotic silencing by unpaired DNA; interacts with other components of the silencing machinery. G3 (Bethesda).

[45] Motamedi M.R., Verdel A., Colmenares S.U., Gerber S.A., Gygi S.P., Moazed D. (2004). Two RNAi complexes; RITS and RDRC; physically interact and localize to noncoding centromeric RNAs. Cell.

[46] Bartel D.P. (2004). MicroRNAs, genomics; biogenesis; mechanism; and function. Cell.

[47] Nicolas F.E., de Haro J.P., Torres-Martinez S., Ruiz-Vazquez R.M. (2007). Mutants defective in a *Mucor circinelloides* dicer-like gene are not compromised in siRNA silencing but display developmental defects. Fungal Genet. Biol.

[48] De Haro J.P., Calo S., Cervantes M., Nicolas F.E., Torres-Martinez S., Ruiz-Vazquez R.M. (2009). A single dicer gene is required for efficient gene silencing associated with two classes of small antisense RNAs in *Mucor circinelloides*. Eukaryot. Cell.

[49] Cervantes M., Vila A., Nicolás F.E., Moxon S., de Haro J.P., Dalmay T., Torres-Martínez S., Ruiz-Vázquez R.M. (2013). A single *argonaute* gene participates in exogenous and endogenous RNAi and controls cellular functions in the basal fungus *Mucor circinelloides*. PLoS One.

[50] Zhang Z., Chang S.S., Zhang Z., Xue Z., Zhang H., Li S., Liu Y. (2013). Homologous recombination as a mechanism to recognize repetitive DNA sequences in an RNAi pathway. Genes Dev.

[51] Yang Q., Li L., Xue Z., Ye Q., Zhang L., Li S., Liu Y. (2013). Transcription of the major *Neurospora crassa* microRNA–like small RNAs relies on RNA polymerase III. PLoS Genet.

[52] Colmenares S.U., Buker S.M., Buhler M., Dlakic M., Moazed D. (2007). Coupling of double-stranded RNA synthesis and siRNA generation in fission yeast RNAi. Mol. Cell.

[53] Verdel A., Jia S., Gerber S., Sugiyama T., Gygi S., Grewal S.I., Moazed D. (2004). RNAi-mediated targeting of heterochromatin by the RITS complex. Science.

[54] White S.A., Allshire R.C. (2008). RNAi-mediated chromatin silencing in fission yeast. Curr. Top. Microbiol. Immunol.

[55] Emmerth S., Schober H., Gaidatzis D., Roloff T., Jacobeit K., Buhler M. (2010). Nuclear retention of fission yeast *dicer* is a prerequisite for RNAi-mediated heterochromatin assembly. Dev. Cell.

[56] Xue Z., Yuan H., Guo J., Liu Y. (2012). Reconstitution of an Argonaute-dependent small RNA biogenesis pathway reveals a handover mechanism involving the RNA exosome and the exonuclease QIP. Mol. Cell.

